# Factors underlying quality problems with alcohol screening in routine care

**DOI:** 10.1186/1940-0640-8-S1-A85

**Published:** 2013-09-04

**Authors:** Emily C Williams, Carol E Achtmeyer, Stacey E Rittmueller, Gwen T Lapham, Laura J Chavez, Rachel M Thomas, Douglas B Berger, Katharine A Bradley

**Affiliations:** 1 Health Services Research & Development (HSR&D), Veterans Affairs (VA) Puget Sound Health Care System, Seattle, WA, USA; 2 University of Washington School of Public Health, Department of Health Services, Seattle, WA, USA; 3 Group Health Research Institute, Seattle, WA, USA

## 

Since 2004 >90% of outpatients in the US Veterans Health Administration (VA) have been screened for unhealthy alcohol use with the AUDIT-C. However, research suggests variability in the quality of screening. To understand factors underlying variable quality, we conducted two qualitative studies: 1) an ethnographic study where we observed clinical staff performing screening, and 2) a key-informant study where we conducted 1:1 interviews with clinical staff. For Study 1, four researchers observed alcohol screening at 9 primary care clinics and took handwritten notes, which were transcribed. For Study 2, snowball sampling was used to recruit key informants (n=29) at 5 additional clinics who completed 20-30 minute semi-structured interviews, which were recorded and transcribed. Both qualitative datasets were analyzed using an *a priori* coding template. In Study 1, we observed 58 clinical staff caring for 166 patients. Alcohol screening was observed 74 times. Clinical staff appeared uncomfortable conducting verbal alcohol screening, and most screening was not verbatim. Study 2 interviews found that clinical staff and providers believed that addressing unhealthy alcohol use is an important part of care but had not received standard training regarding how or why to conduct alcohol screening. Information on alcohol screening was provided to clinicians via email announcement of the availability of electronic clinical decision support and ad-hoc peer-to-peer demonstration of its use. Participants perceived the screening questions to be sensitive and reported modifying questions to increase patient comfort. Participants were largely focused on identifying patients with the most severe condition—alcohol dependence—for which brief intervention does not have confirmed efficacy. Lack of training and discomfort are barriers contributing to variability in screening quality. Addressing the spectrum of unhealthy alcohol use is not yet viewed as part of a preventive agenda. Additional strategies are likely needed to improve screening quality.

